# A multimetric approach: Utilizing diatom communities to assess acid mine drainage impacts on South African wetlands

**DOI:** 10.1007/s10661-026-15534-4

**Published:** 2026-06-12

**Authors:** Ruan C. J. Koen, Melanie de Nysschen, Jonathan C. Taylor

**Affiliations:** 1 Unit for Environmental Science and Management, North-West University (NWU), Private Bag X6001, Potchefstroom, 2520 South Africa; 2 South African Institute for Aquatic Biodiversity (SAIAB), Private Bag 1015, Makhanda/Grahamstown, 6140 South Africa

**Keywords:** Bioassessment, Bacillariophyceae, Diatom indices, Community weighted metrics, Wetlands, Environmental disturbance

## Abstract

Acid mine drainage (AMD) is a major concern for wetlands located in coal-bearing regions, particularly where mining activities and associated spoil material are present. In this study, a diatom-based index is proposed to detect acidification related to AMD in wetlands surrounding coal mines, with a specific focus on the principal environmental stressors associated with AMD: low pH, low alkalinity, elevated electrical conductivity (EC), and elevated sulphate concentrations. Chloride was included as a secondary indicator reflecting mine-related ionic enrichment rather than direct AMD-generating processes. A total of 87 samples were collected from 45 sites across two seasons (dry and wet) to capture spatial and temporal variation in diatom community structures. Epiphytic diatoms were sampled using standard protocols, and species composition was derived alongside environmental variables. This dataset formed the basis for multivariate analyses linking diatom community structure to environmental gradients associated with acidification. Canonical correspondence analysis (CCA) demonstrated that diatom species distributions were primarily structured along an acidification gradient driven by pH (−0.93), alkalinity (−0.79), and sulphate (0.65), with a secondary ionic gradient associated with chloride and EC. Weighted averaging (WA) was used to calculate environmental optima for species under key stressors, enabling diatom taxa to be positioned along ecological gradients. These optima were used to calculate community-weighted metrics (CWMs), which were integrated into two multimetric indices (MMIs). Redundancy analysis (RDA) of the derived indices revealed strong and consistent associations with the same environmental gradients identified in the CCA, pH (−0.91), sulphate (0.67), and alkalinity (−0.78), and clearly distinguished between acidified and non-acidified wetlands. The correspondence between CCA and RDA results indicates a hierarchical compression of ecological information from species assemblages to indices while retaining dominant environmental gradients, making the proposed MMI a practical tool for distinguishing wetland acidification under AMD influence where routine water quality monitoring is constrained.

## Introduction

Wetlands, as defined by the South African National Water Act (NWA, 1998), are transitional areas between land and water where the water table is near the surface or the land is periodically submerged, supporting vegetation adapted to saturated soils. In South Africa, wetlands are classified as aquatic systems due to the periodic presence of water (Ollis et al., 2013). They provide important ecosystem services including flood attenuation and water purification, while also supporting high biodiversity and many endemic species (Collins, 2005).

National assessments indicate that 79% of the country’s 135 inland wetland ecosystem types are threatened, with 62% critically endangered, 9% endangered, and 9% vulnerable, while only 21% are of least concern. In addition, 61% of inland wetland types are not protected, with only 6% well protected (van Deventer & Nel, 2025).

Wetlands are increasingly impacted by anthropogenic pressures, including industrial pollution and acid mine drainage (AMD). Coal mining exposes sulphur-bearing minerals such as pyrite, which oxidize in contact with water to form acidic effluent (Ochieng et al., 2010). Wetlands, as natural sinks for water, nutrients, and salts, readily receive this runoff. AMD is characterized by elevated sulphate concentrations, increased electrical conductivity (EC), and reduced pH (Smucker & Vis, 2009). Although wetlands have natural buffering and assimilative capacity, this can be exceeded under sustained AMD pressure, leading to ecological degradation (Humphries et al., 2017).

Biomonitoring is widely used in South Africa to assess aquatic ecosystem condition through physical, functional, and biotic indicators (Kotze et al., 2012). Biological assessments typically rely on changes in species composition and abundance, with diatoms, macrophytes, macroinvertebrates, and fish serving as established bioindicators (Dallas et al., 2010; Kleynhans, 1999). Diatoms are particularly effective due to their rapid reproduction, environmental sensitivity, and wide distribution (Hattikudur et al., 2015; Lobo et al., 2016). Their ecological preferences for pH, conductivity, nutrients, and organic content are well documented, and their short life cycle (~ 2 weeks) enables rapid response to disturbance. Acid-tolerant taxa further enhance their value in AMD-impacted systems (Battarbee et al., 2004; Harding et al., 2005).

Diatom community structure, including growth-forms, life-forms, and ecological guilds, provides additional ecological insight. These reflect adaptations related to morphology, mobility, and resource use, and are grouped into solitary, motile, attached, and colony-forming forms, as well as low-profile, high-profile, motile, and planktonic guilds (Hudon & Legendre, 1987; Rimet & Bouchez, 2012).

A diatom-based multimetric index for AMD-impacted wetlands was developed by Riato et al. (2017) and later tested by Erasmus (2021). This index used biological metrics such as similarity to reference conditions, functional composition, and taxonomic diversity, but did not include chemical variables. As a result, AMD impacts are inferred indirectly through biological change rather than measured along environmental gradients.

In contrast, the present study quantifies AMD pressure directly using diatom ecological optima derived from measured chemical variables (pH, sulphate, EC, alkalinity, and chloride), combined into a weighted multimetric index. This allows application across multiple wetland types, including seeps, floodplain wetlands, and valley-bottom wetlands, rather than being restricted to depressional systems.

Riverine diatom indices adapted from European systems are often unsuitable for wetlands due to fundamental ecological differences. Wetlands are dynamic systems at the terrestrial–aquatic interface, often with high nutrient and organic loads, and function as sinks for sediments and pollutants (Fisher & Acreman, 2004). As a result, riverine indices may misclassify natural wetland conditions as impacted.

AMD impacts further complicate assessment because wetlands vary in buffering capacity. When exceeded, AMD leads to acidification, increased metal solubility, and reduced ecosystem resilience (Tatu et al., 2008; Dean et al., 2013). Coal-derived AMD is typically dominated by sulphate and sulphide, while gold/uranium mining introduces higher heavy metal loads and long-term toxicity (Laker, 2023; Mogashane et al., 2025).

Key AMD-related variables include sulphate, pH, alkalinity, chloride, and EC. Sulphate increases with AMD, while pH, alkalinity, and chloride generally decrease, and EC increases. Chloride primarily reflects natural weathering and external inputs, while sulphate to chloride ratios help distinguish pollution sources (Humphries et al., 2017). These five variables form the basis of the community-weighted metrics used in the multimetric index developed in this study.

This study aims to identify diatom community structures in healthy wetland systems with intact buffering capacity and in wetlands where acidification has exceeded buffering capacity, in order to develop a multimetric index capable of detecting persistent acidification related to acid mine drainage (AMD), using species optima derived from both acidic and non-acidic (reference) wetland conditions.

## Methodology

**Fig. 1:**
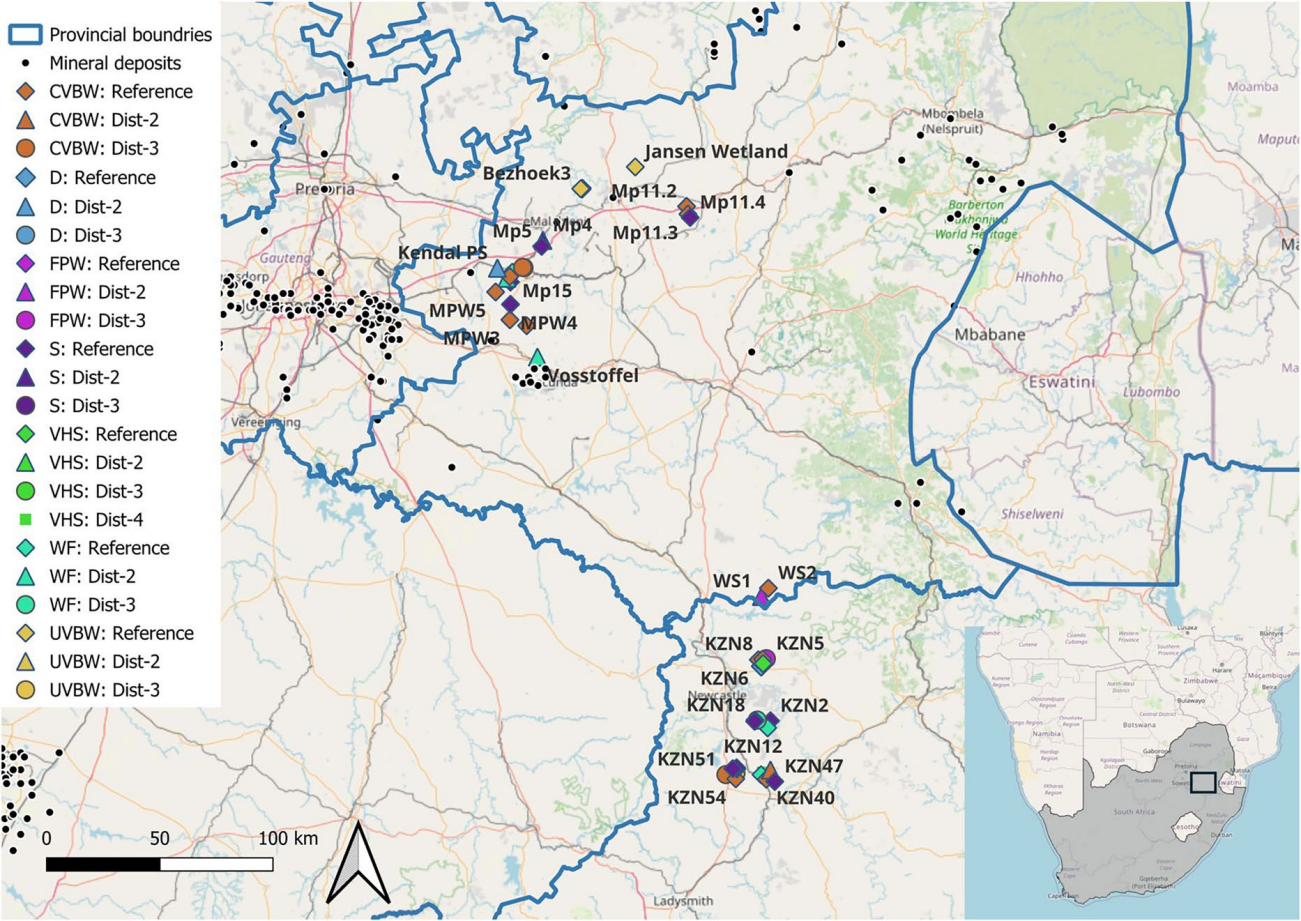
Locations of sampling sites in Mpumalanga (top) and KwaZulu-Natal (bottom). Sites are coloured based on wetland type (Appendix Table 4). Symbols represent reference sites and disturbed sites sampled during the dry season

**Fig. 2:**
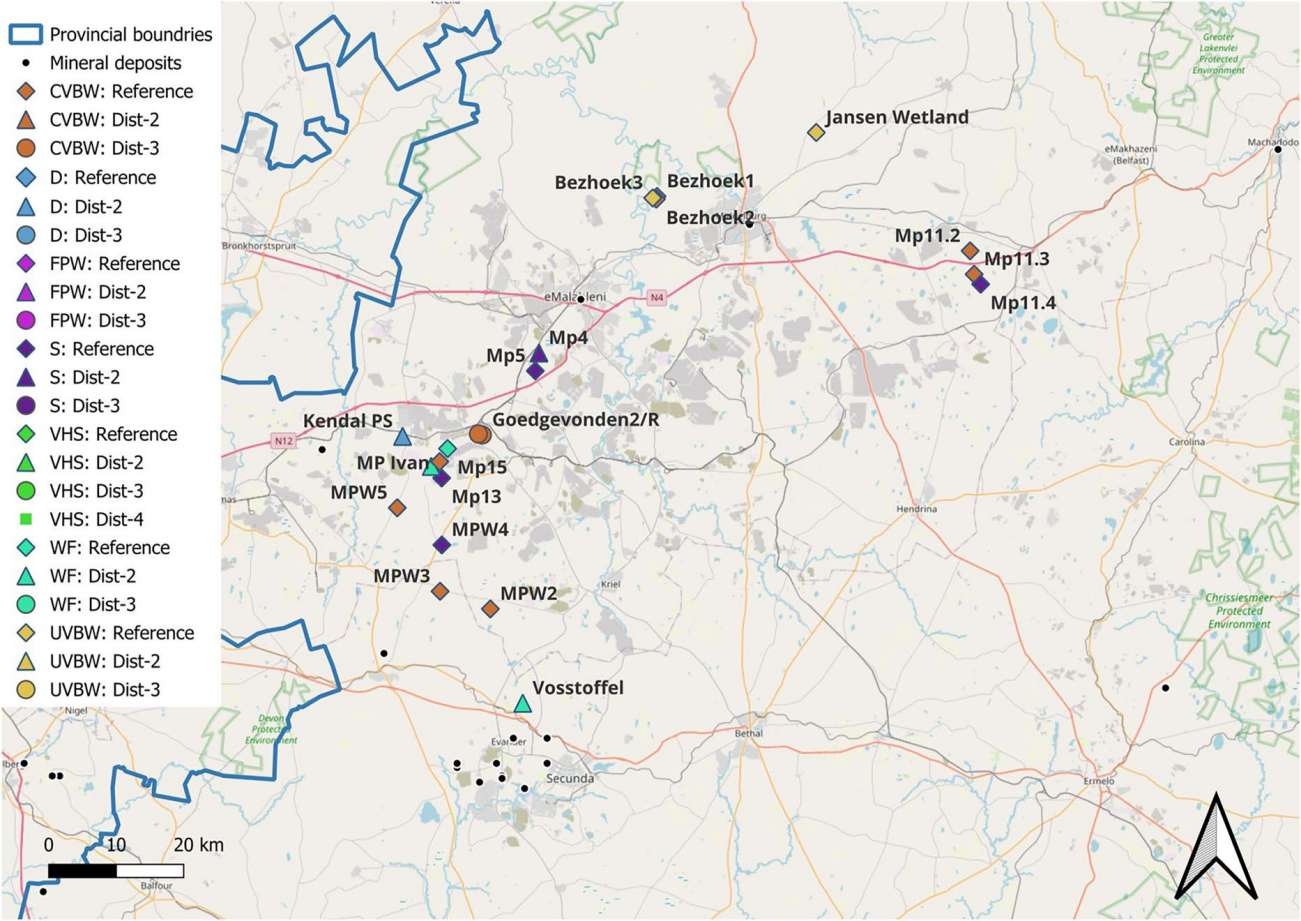
Locations of sampling sites in the Mpumalanga province. Grey areas on the map indicate mining activity

**Fig. 3:**
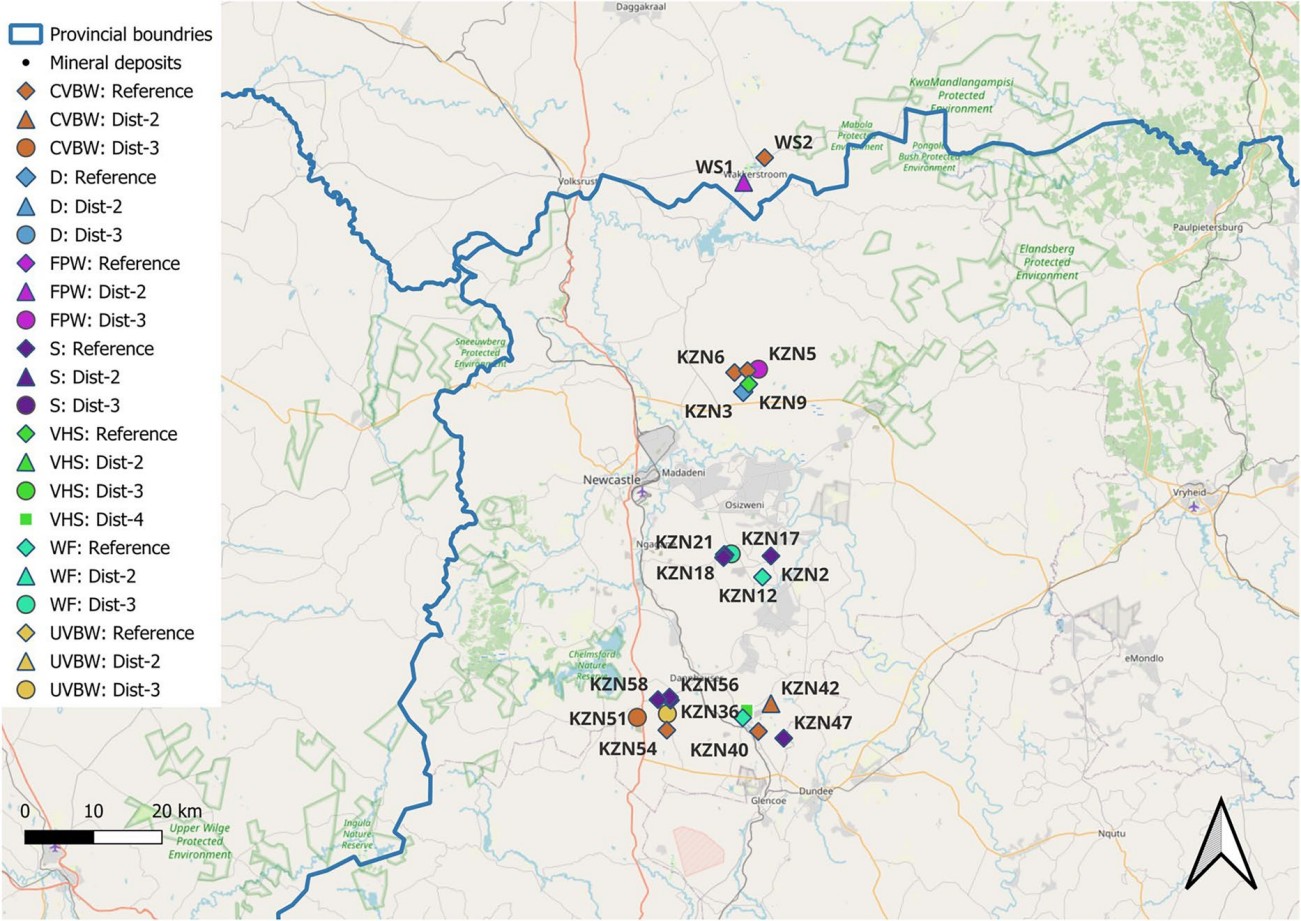
Locations of sampling sites in the KwaZulu-Natal province. Grey areas on the map indicate mining activity

A dataset containing data from eighty-seven samples taken from 45 distinct wetland sites (Fig. 1) with corresponding diatom abundances and measured environmental parameters was used to create the multimetric index. Sites were selected to include reference sites (reference) as well as disturbed sites (D-2, D-3 and D-4). Disturbance status, classified as acidification and loss of buffering capacity, was initially inferred based on site proximity to mining activities and subsequently confirmed using water quality data, with particular emphasis on measured sulphate concentrations, pH and EC (Appendix Table 3). Based on water quality, only a few sites are affected by AMD (pH < 5 and/or sulphate > 800 mg/L and/or EC > 800 mg/L), other sites represent healthy wetlands systems with a functional buffering capacity. Where possible, each site was sampled twice for diatoms and water quality, once during the dry season (all 45 sites) and once during the wet season (42 of the 45 sites). A total of eighty-seven samples were collected across both seasons (July 2022 (dry) and February 2023 (wet)). Two separate geographical areas were selected, twenty-one sites were sampled in the eMalahleni area in Mpumalanga (Fig. 2) and twenty-four in the New Castle area in KwaZulu-Natal (Fig. 3).

Epiphytic diatoms were sampled according to Taylor et al. (2007a). A minimum of five sublocations were sampled in duplicate in each wetland and combined to represent the diatom community structure of each site. Plant material was scraped with a toothbrush, and a concentrated diatom sample was poured into a 50 ml sampling vial and preserved with 70% ethanol. Diatom samples were processed according to Taylor et al. (2007a) with acid digestion using the hot HCl/H_2_O_2_ method. Samples were cleaned and dried on coverslips before permanent mounting with Pleurax (Von Stosch, 1974). At least 400 diatom frustules were counted for each site and taxa were identified to species level using a Nikon 80i microscope under 1000× magnification with Differential Interference Contrast (DIC) using relevant taxonomic literature. Diatom taxa were identified using standard morphological criteria (valve shape, striae pattern, and raphe structure) and taxonomic keys provided in the *Süßwasserflora von Mitteleuropa* series (Krammer & Lange-Bertalot, 1986–1991), relevant monographs from *Diatoms of Europe* (Lange-Bertalot, 2000–present), and the regional guide of Taylor et al. (2007b). These sources provide detailed species descriptions, illustrations, and identification keys for freshwater diatoms. The inclusion of both European and regional literature allowed for consistent identification while accounting for morphological variability in southern African taxa. More recent taxonomic revisions were consulted where necessary to ensure up-to-date nomenclature.

The environmental variables were measured *in situ* using a Horiba LAQUA-twin test kit (EC, Na^+^, K^+^, NO_3_^2−^,Ca^+^, and pH) and a Hanna multi-oxygen meter (temperature and dissolved oxygen) and *ex situ* through laboratory analysis at the SASNAS accredited laboratory of the Council for Scientific and Industrial Research (CSIR) in Stellenbosch, South Africa. Only the water quality variables measured during laboratory analysis were used for statistical analyses to ensure standardization of results.

Canonical correspondence analysis (CCA) was done using CANOCO v4.51 to explore the species-environment relationship of diatom communities to determine if the diatom community structure is correlated to environmental variables measured before proceeding with the index calculation. CCA analysis is a powerful ordination technique that allows the illustration of site, species and environmental variable relationships. CCA assumes a direct unimodal relationship between environmental variables and species data at sample sites. Therefore, the change in diatom community structure across sites can be illustrated with the corresponding environmental variables at sites. A Monte-Carlo permutation test was applied, with 999 permutations, to test the overall significance of the species-environment relationship. Forward selection was used to identify which environmental variables serve as significant predictors of diatom community composition.

An RDA ordination using CANOCO v4.51 was done to illustrate the relationship between index scores, environmental variables and sites. The five environmental variables used to create the index were used in the RDA to determine how well the calculated scores correlated to the environmental variables associated with AMD impact. An RDA was chosen based on the linear scale of index scores and environmental variables, before the ordination was done environmental data was log transformed.

Before calculating the MMI index, the optimal environmental conditions (*µ*) of taxa were estimated for pH, sulphate, EC, alkalinity, and chloride. To estimate optimal environmental conditions, taxa were required to occur at a minimum of 10 sites with a relative abundance exceeding 1%. This study-specific threshold was selected to balance the inclusion of informative taxa with the exclusion of rare occurrences that may yield unreliable optima. Of the 290 taxa identified, a total of 78 (26.9%) satisfied this criterion and a ***µ***-value for each was estimated through weighted averaging (WA) (Ter Braak & Barendregt, 1986).

It is important to note that species optima were calculated using the original relative abundances derived from the full dataset. Relative abundances were not rescaled after reducing the dataset to the selected seventy-eight species. As a result, the relative abundance values used in the analysis continue to reflect the true proportional representation of each species within the original diatom communities. This approach ensures that species-environment relationships are based on ecologically realistic community structure.

The optimum environmental condition (***µ***) estimation is based on the following formula:

1 $${{\boldsymbol{\mu}}}_{k}= \sum_{i=1}^{n}{Y}_{ik}{X}_{i}/\sum_{i=1}^{n}{Y}_{ik}$$

where *Y*_*ik*_ is the abundance of the *k*-th taxon in the *I*-th sample and *X*_*i*_ is the environmental variable value in the *I*-th sample. Using this approach, weighted average (WA) optima were estimated for each diatom taxon separately for each environmental stressor associated with AMD.

Diatoms fill functional niches within their community and either facilitate or hinder the growth of other diatom species in the community. Diatom species also have physiological adaptation to thrive under varying degrees of nutrient concentrations, organic pollution, and light availability (Patrick, 1977). However, this is largely accounted for through calculation of the optimum environmental condition.

The calculation of the multimetric index is as follows: For each environmental stressor, a community-weighted metric (CWM) was calculated using the weighted-average optima of diatom taxa present at each site:

2 $${CWM}_{i}= \sum_{k=1}^{n}{\mu}_{k}{a}_{k}/\sum_{k=1}^{n}{a}_{k}$$

where ${CWM}_{i}$ is the community-weighted metric for environmental stressor $i$, ${\mu}_{k}$ is the estimated optimum value of parameter $i$ for the $k$-th diatom taxon, ${a}_{k}$ is the relative abundance of the $k$-th taxon, and $n$ is the total number of taxa present at the site. This calculation produces a single community-weighted value for each environmental stressor for each site. The procedure was repeated for all five selected parameters, resulting in five CWMs per site.

To place all CWMs on a common scale, values were normalized using the observed minimum and maximum CWM values across all sites:

$${CWM}_{norm}= ({CWM}_{i }-{CWM}_{min}/{CWM}_{max }- {CWM}_{\mathrm{m}\mathrm{i}\mathrm{n}})*100$$

For parameters where lower values indicate stronger AMD impact (pH and alkalinity), the normalized CWMs were directionally reversed using $100-{CWM}_{norm}$. This reversal was applied after normalisation but prior to the assignment of variable weights and index aggregation. Applying the reversal at this stage represents the earliest point at which all variables are on a common, information-preserving scale, thereby ensuring directional consistency while avoiding potential distortion or information loss during subsequent weighting and transformation steps.

Two index formulations were developed. The first applied an equal weighting of CWMs, allowing all CWMs to contribute equally to the index score (MMI 1). This serves as a baseline for further comparison. The second applied differential weighting with more emphasis on sulphate, pH and EC than on alkalinity and chloride (MMI 2). This reflects the primary chemical characteristics of AMD and a stronger ecological response of diatoms to these stressors. Using both approaches allows comparison between general water quality and AMD-specific stressors.

The multimetric indices (MMIs) were calculated as a weighted sum of the normalized and directionally aligned CWMs:

$$MMI=\left({CWM}_{sulp}\times {W}_{sulp}\right)+\left({CWM}_{pH}\times {W}_{pH}\right)+ \left({CWM}_{EC}\times {W}_{EC}\right)+ \left({CWM}_{chl}\times {W}_{chl}\right)+ \left({CWM}_{alk}\times {W}_{alk}\right)$$

In the case of MMI 1, all CWMs were assigned equal weights of 0.2. In the case of MMI 2, the weights applied to each parameter were: sulphate = 0.30, pH = 0.25, EC = 0.15, chloride = 0.10, and alkalinity = 0.20.

To assess the reliability of the calculated index scores, bootstrapping was performed in the absence of additional datasets. One thousand iterations of the index calculation were carried out by resampling the optimal environmental conditions for each parameter, with each value adjusted by 2% of its respective range. The resulting index scores were then validated using Pearson correlations to confirm the consistency of the index.

The index calculation process has been streamlined through the development of a user-friendly executable software program. The executable program was developed in Python and compiled as a standalone Windows application. Users input species and environmental data and perform the index calculation. The program automatically matches the provided species names to a standardized list containing the optimal environmental condition for each taxon for each of the principal environmental stressors. Once matched, the program performs the necessary calculations and generates an Excel spreadsheet listing each site from the input file along with its calculated index score for easy comparison and interpretation.

## Results and discussion

### Canonical correspondence analysis (CCA)

**Fig. 4:**
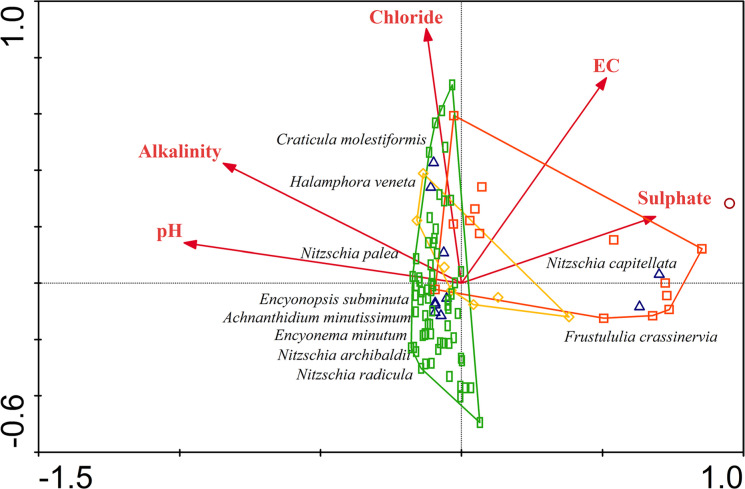
CCA illustrating the relationship between species and environmental variables (red arrows) at sites sampled (geometric shapes). The convex hulls represent observed disturbance levels of sites (green rectangles—low disturbance, yellow diamonds—moderate disturbance, orange squares—high disturbance, red circle—extreme disturbance). Blue triangles represent species

From the CCA analysis (Fig. 4), pH (−0.93), alkalinity (−0.79), and sulphate (0.65) are the main driving factors influencing the species distribution on the first axis, that suggests species are separated along the pH, sulphate gradient. The second axis represents the chloride and EC gradient, suggesting this gradient is a secondary driving factor in the species distribution with chloride having a strong correlation with the second axis (0.76) and EC having a slightly weaker relationship (0.61). The strong separation of sites along axis one by pH, alkalinity, and sulphate indicates the diatom community structure is primarily structured by an acidification gradient rather than by ionic enrichment alone. This is supported by multiple studies that show how diatom community structures are primarily separated along pH gradients (Battarbee et al., 2004; Smol & Stoermer, 2010) and that pH affects metabolic processes and structural development of diatoms (Hervé et al., 2012; Lunay et al., 2020).

The global Monte Carlo permutation confirmed that the full set of environmental variables significantly explained variation in species composition (Trace = 1.888, *F* = 1.849, *p* = 0.001). The constrained ordination explained 42.8% of the species-environment relationship on the first axis and 70.9% cumulatively across the first two axes, with eigenvalues of 0.809 (Axis 1), 0.530 (Axis 2), 0.320 (Axis 3), and 0.151 (Axis 4), indicating that most explained variation was concentrated in the first two gradients. Forward selection identified pH as the most significant predictor of community composition (*p* = 0.002), followed by chloride (*p* = 0.014), while EC showed a weaker, marginal effect (*p* = 0.086). Alkalinity (*p* = 0.154) and sulphate (*p* = 0.978) did not contribute significantly once other variables were included in the model. Interestingly sulphate was not identified as a significant factor according to the forward selection, this is perhaps due to the collinearity with pH and EC, which suggest sulphate is not an independent predictor but rather part of a broader hydrochemical gradient. Overall, Axis 1 represents a dominant hydrochemical gradient strongly correlated with pH (*r* = −0.91) and associated with increasing ionic content (EC and sulphate), reflecting a shift from less mineralised, higher pH conditions to more ion-rich and lower pH environments. This highlights the importance of using multivariate approaches to index development, as using single parameters in index calculation may underestimate ecological influences.

Figure 4 also illustrates taxa contributing to more than 8% of the community structure. *Nitzschia capitellata* and *Frustulia crassinervia* are strongly associated with low pH, increased sulphate, increased EC, and decreased alkalinity. These taxa also show a weaker association with chloride concentrations. The associations of these taxa indicate they prefer acidic environments with high sulphate concentrations. Taxa showing a strong association with increased chloride and moderate-to-high EC are *Halamphora veneta* and *Craticula molestiformis*. This suggests these taxa tolerate high EC environments with higher chloride concentrations. They are also associated with neutral pH and slightly higher levels of alkalinity.

Other taxa present (*Nitzschia* *palea*, *Encyonopsis subminuta*, *Achnanthidium minutissimum*, *Encyonema minutum*, *Nitzschia archibaldii*, and *Nitzschia radicula*) are abundant in sites with neutral pH and alkalinity, moderate sulphate and moderate conductivity and lower chloride concentrations. As the sulphate concentrations increase above 800 mg/L and the pH decreases below 3, these taxa disappear from the community, *Nitzschia capitellata* and *Frustulia crassinervia* become more abundant and dominate the community in disturbed conditions. As the chloride concentration and EC increase, the former taxa also disappear from the community and are replaced by *Craticula molestiformis* and *Halamphora veneta* which become highly abundant.

From the results, unimpacted wetlands (intact buffering capacity) were dominated by *Achnanthidium minutissimum*, *Encyonopsis subminuta*, *Encyonema minutum*, *Nitzschia archibaldii*, *Nitzschia radicula*, and *Nitzschia* *palea*. These species share a diversity of life-forms (motile, attached, and tube-forming). As water quality shifts to higher sulphate concentrations, low pH, low alkalinity and elevated EC, the community structure shifts to one dominated by *Nitzschia capitellata* and *Frustulia crassinervia*. This suggests that these species are indicators of acidification in wetlands as their dominance in the community structure correlates with observed disturbance levels. These species are motile and are known to occur under highly acidic environments (De Nicola, 2000; Kulichová & Fialová, 2016; Passy, 2007). Physiological adaptations in *Nitzschia capitellata* and *Frustulia crassinervia* under low pH give them a competitive advantage under reduced buffering conditions which support their suitability as indicator taxa of acidification. Furthermore, their presence not only reflects chemical gradients but also a restructuring of the community.

As water quality shifts to higher chloride concentrations and elevated EC, the community structure is largely dominated by *Halamphora veneta* and *Craticula molestiformis* which are small, motile species. This suggests that these species are indicative of unimpacted wetlands with increased chloride concentrations and are known to occur in electrolyte-rich waters (Taylor et al., 2007b). The taxa that dominate the community structure are therefore a mix of different life forms; however, motile species are present in all disturbance levels, suggesting that life form is not a diagnostic trait that can be used to assess acidification in wetlands, rather, the occurrence of these species is likely linked to physiological adaptations. It is also worth noting that diatom teratologies were not considered in this study but could be an important morphological feature to consider in future studies as they are linked to metal stress and can be indicators of acidification (Olenici et al., 2017; Walsh & Wepener, 2009).

The CCA was therefore useful in indicating how diatom community structure alters as the environment changes. With prolonged acidification the diatom community structure shifts from a community dominated by *Achnanthidium minutissimum*, *Encyonopsis subminuta*, *Encyonema minutum*, *Nitzschia archibaldii*, *Nitzschia radicula*, and *Nitzschia* *palea* to a community dominated by *Nitzschia capitellata* and *Frustulia crassinervia*. Calculating species optima for diatom species for these environmental parameters and including them in a multimetric index will accurately reflect the environmental change associated with acidification caused by AMD.

### Optimal environmental conditions

Using weighted averaging, optimum environmental conditions for taxa were calculated. Five key taxa with a strong relationship to AMD disturbance in wetlands were identified using CCA (Appendix Table 1). These taxa are *Nitzschia capitellata*, *Frustulia crassinervia, Encyonopsis microcephala*, *Nitzschia pura*, *Craticula buderi*, and *Navicula veneta*. These taxa have optimum environmental conditions for EC above 1000 µS/cm, sulphate above 350 mg/L, chloride below 150 mg/L, and alkalinity below 85 mg/L. Additionally, the pH value for *Nitzschia capitellata* and *Frustulia crassinervia* as calculated through weighted averaging was below 4, which indicates these species are correlated with low pH, high sulphate concentrations, high EC, low alkalinity and low chloride concentrations. However, of these five taxa only *Frustulia crassinervia* and *Nitzschia capitellata* represent more than 8% of the community structure, therefore these species serve as indicators for acidification in wetlands, where the other three species may serve as potential indicators but do not dominate in acidic conditions.

Taxa serving as strong indicators for undisturbed wetlands (existing buffering capacity) are *Navicula* *arvensis* var. *maior*, *Halamphora veneta*, *Craticula molestiformis*, *Nitzschia* *amphibia*, and *Mayamaea atomus* These taxa all have optimum environmental conditions for pH above 8.5, alkalinity above 130 mg/L and chloride above 70 mg/L. However, only *Halamphora veneta* and *Craticula molestiformis* represented more than 8% of the community structure*.*

Therefore, *Nitzschia capitellata*, *Frustulia crassinervia, Halamphora veneta*, and *Craticula molestiformis* can serve as reference taxa for rapid assessment of the presence or absence of acidification prior to index calculation.

### MMI score analysis

**Fig. 5:**
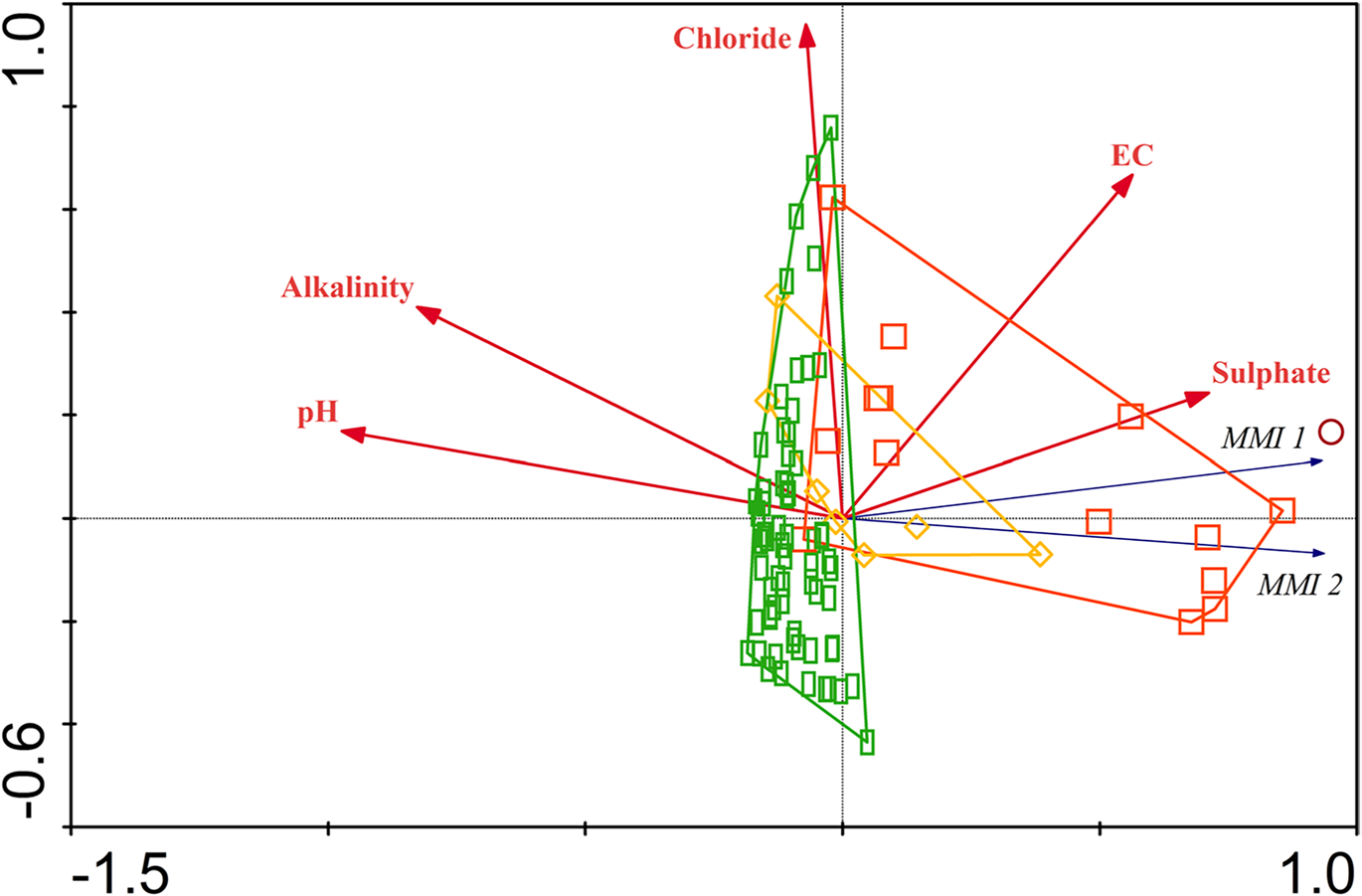
RDA of index scores (blue arrows), environmental variables (red arrows) and sites (shapes). Sites are grouped based on observed disturbances (green rectangles—low disturbance, yellow diamonds—moderate disturbance, orange squares—high disturbance, red circle—extreme disturbance)

The RDA (Fig. 5) shows an increase in the index scores (MMI 1 & 2) with decreasing pH and alkalinity, and increasing sulphate and EC. MMI 1 follows the above methodology with equal weighting of environmental variables, whilst the MMI 2 follows the same method with differential weighted environmental variables; the purpose of this approach is to demonstrate how much influence weighting environmental parameters has on the final index score and correlation. Chloride has a weak positive relationship with the calculated index scores. The index scores also have a positive relationship with disturbance groupings, as the index scores increase, the disturbance level increases. MMI2 has a stronger positive relationship with pH and alkalinity, while the MMI 1 has a stronger positive relationship with sulphate. The RDA shows that pH, alkalinity and sulphate concentrations are correlated strongly with index scores, as pH and alkalinity decrease the index score increases and as sulphate increases, the index scores increase accordingly.

According to the RDA, the first axis (*x*-axis) explains 94% of the species-environment relationship and explains 56.7% of the variance in the species data. The first axis explains most of the species environment relationship and is strongly correlated with pH (−0.91), alkalinity (−0.78), and sulphate (0.67). Therefore, the analysis would suggest that the species distribution in sites is highly correlated with pH, alkalinity, and sulphate.

**Fig. 6:**
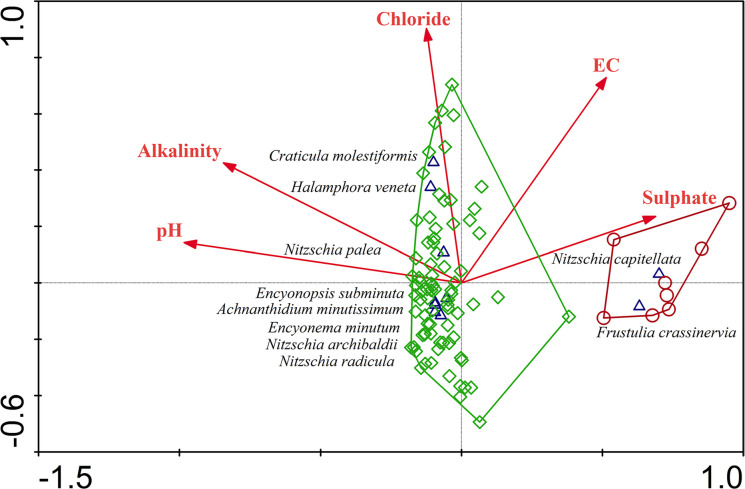
CCA of diatom community structure and measured water quality, with convex hulls representing site groupings based on disturbance levels derived from MMI 2 (reference sites—green diamonds; disturbed sites—red circles)

**Fig. 7:**
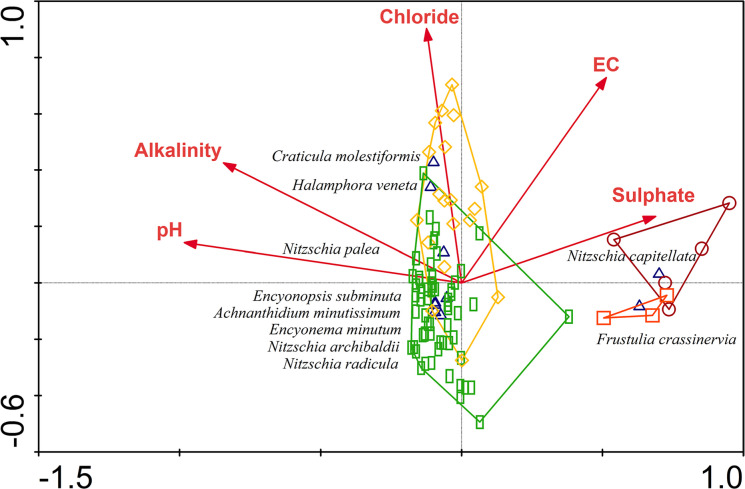
CCA of diatom community structure and measured water quality, with convex hulls representing site groupings based on disturbance levels derived from MMI 1 (reference sites—green rectangles; disturbance level 1 sites—yellow diamonds; disturbance level 2 sites—orange squares; disturbance level 3 sites—red circles)

The same CCA ordination in Fig. 4 was used to illustrate how disturbance classes calculated from the MMI scores separate sites along an environmental gradient in Figs. 6 and 7. Disturbance levels were defined using quartiles of the index scores, with sites below 25 classified as reference, 25–50 as disturbance level 1, 50–75 as disturbance level 2, and above 75 as disturbance level 3 (Appendix Table 2). Figure 6 illustrates the separation of sites based on MMI 2. Sites are classified as either reference (green diamonds) or disturbed (red circles). Disturbed sites are characterised by elevated sulphate and EC, along with low pH and alkalinity. Reference sites have moderate sulphate, EC, pH, and alkalinity, and exhibit higher chloride levels than disturbed sites. Only eight sites were classified as disturbed, while 79 sites were classified as reference. Using MMI 1 (Fig. 7), a clearer separation of disturbance levels is observed, more closely matching the patterns of observed disturbance. Unlike the observed disturbance levels, MMI 1 provides a clearer distinction between reference and disturbance level 1 sites versus disturbance levels 2 and 3, indicating that MMI 1 is more effective in distinguishing wetland disturbance based on diatom-inferred water quality than MMI 2. Therefore, using equal weighting of CWMs provides a clearer picture of the multifactorial acidification in these wetlands and indicates that acidification is not driven by a single dominant variable but by the combined influence of multiple interacting variables.

The strong correspondence between the RDA and CCA ordinations indicates that the ecological structure observed in species space is preserved through the successive transformation steps. The dominant gradients identified in the CCA are retained and re-expressed in the RDA of index scores, demonstrating that the multimetric index functions as an ecological compression of the original community-environment relationships.

### Bootstrapping

The reliability and efficiency of indices are validated using independent data or resampling methods (bootstrapping). The calculated optimal environmental conditions for each environmental variable were bootstrapped with error values of 2% of the entire range of measured values and done accordingly: sulphate—70 mg/L; chloride—6.5 mg/L; alkalinity—6.5 mg/L, EC—150 µS/cm, and pH—0.15. Values below zero were adjusted to 0.1. A total of 1000 bootstrapping iterations were performed, after which Pearson correlations were calculated for the newly derived indices. For MMI 1, the average correlation for sulphate was 0.69 with a 95% confidence interval of [0.690, 0.693], chloride was −0.04 with a 95% confidence interval of [−0.038, −0.043], alkalinity was −0.72 with a 95% confidence interval of [−0.727, −0.725], EC was 0.6 with a 95% confidence interval of [0.598, 0.601], and pH was −0.89 with a 95% confidence interval of [−0.890, −0.888]. For MMI 2, the average correlation for sulphate was 0.67 with a 95% confidence interval of [0.670, 0.673], chloride was −0.045 with a 95% confidence interval of [−0.048, 0.043], alkalinity was −0.765 with a 95% confidence interval of [−0.770, −0.767], EC was 0.544 with a 95% confidence interval of [0.542, 0.546], and pH was −0.913 with a 95% confidence interval of [−0.914, −0.912]. The correlations of the index scores with measured water quality variables remained strong and consistent, especially for pH, alkalinity, and sulphate, indicating a high level of reliability in the index calculation. The narrow intervals across iterations indicate species optima are stable under small variations of environmental gradients, suggesting the ecological niches derived are not ecological noise but a reflection of a robust species-environmental relationship.

## Conclusions and recommendations

Attempts to use diatoms as biomonitoring indicators for AMD disturbance in wetlands were initially not particularly successful, largely due to the use of riverine indices in wetland systems without any modification to the calculation of indices. Studies by Riato (2017) have attempted to create a multimetric diatom index for use in wetlands and have been largely successful, however, while Riato (2017) successfully developed a multimetric diatom index for wetlands, it relies solely on biological metrics derived from diatom assemblages to infer AMD impacts. Their approach may not capture the primary chemical drivers of AMD, such as elevated sulphate and low pH, limiting its sensitivity and specificity for AMD detection. Additionally, the calculation of the index is extremely time consuming. Their method provides accurate results; however, the complex nature of the index requires users to have in-depth knowledge of the structure of diatom communities, statistical analysis, and mathematical formulas. The use of this method by consultants and others involved in the routine monitoring of water resources is therefore not feasible as a reliable, quick way to determine AMD impacts in wetland systems.

Keeping in mind the complexity of multimetric indices and the importance of accurate, easily obtainable results, the present study addressed these issues by developing a multimetric index that leverages diatom-inferred water quality, integrating chemical and environmental metrics directly associated with AMD (pH, sulphate, EC, alkalinity, chloride), rather than relying on biological assemblage structure alone. Therefore, using diatoms to determine acidification in wetlands using water quality and not biological metrics derived from the diatom assemblage structure. CCA illustrated the distribution of diatom communities under water quality with a strong association with pH, sulphate and alkalinity. The RDA plots illustrate how the ecological information linking diatom communities to water quality is not lost when calculating the MMIs but rather retained. Therefore, the proposed MMIs provide a practical tool for distinguishing AMD-impacted from unimpacted wetlands, in terms of acidification, where routine water-quality monitoring is constrained. The index calculation is still complex; however, the calculation is housed in an executable computer programme where index scores are automatically calculated from an input sheet, significantly reducing the expertise and time required compared to the method of Riato et al. (2017).

Therefore, the proposed index could be valuable to the Department of Water and Sanitation (DWS), the South African National Biodiversity Institute (SANBI) and provincial management authorities and consultancy companies that assess wetlands impacted by mining activities, by providing an easy-to-use platform that can increase data generation and throughput, and thereby supporting wetland health and rehabilitation, and policy implementation.

The resulting index provides a practical, automated tool for distinguishing acidified from non-acidified wetlands, reducing analytical complexity while maintaining ecological interpretability. This enhances its applicability for routine monitoring by the Department of Water and Sanitation (DWS), the South African National Biodiversity Institute (SANBI) and provincial management authorities and consultancy companies that assess wetlands impacted by mining activities. Future work should expand spatial and temporal datasets to refine species optima and improve the transferability of the index across broader wetland types and mining-impacted regions.

## Data Availability

The datasets generated during the current study are available from the corresponding author upon reasonable request, subject to any applicable data-sharing agreements.

## Appendix

**Table 1:** The optimum environmental conditions for 78 diatom taxa for EC, pH, sulphate, chloride, and alkalinity

Taxon	EC (µS/cm)	pH	Sulphate (mg/L)	Chloride (mg/L)	Alkalinity (mg/L)
*Achnanthidium minutissimum* (Kützing) Czarnecki	312.02	8.7	75.97	9.83	54.31
*Achnanthidium standeri* (Cholnoky) J.C. Taylor, E. Morales & Ector	178.7	8.33	35.87	6.91	37.55
*Adlafia bryophila* (J.B. Petersen) Lange-Bertalot	154.1	7.54	17.9	8.58	41.89
*Aulacoseira granulata* (Ehrenberg) Simonsen	231.45	8.36	33.12	12.56	56
*Aulacoseira granulata* var. *angustissima* (O.Müller) Simonsen	162.36	8.45	29.03	8.46	31.52
*Brachysira neoexilis* Lange-Bertalot	425.87	7.85	150.45	8.92	43.54
*Caloneis bacillum* (Grunow) Cleve	415.56	7.99	82.98	14.22	101.22
*Cocconeis placentula* var. *placentula* Ehrenberg	190.36	8.64	10.79	14.07	58.55
*Craticula buderi* (Hustedt) Lange-Bertalot	1627.67	8.39	566.63	152.99	77.91
*Craticula molestiformis* (Hustedt) Mayama	860.46	9.39	47.62	123.66	180.99
*Stephanocyclus meneghinianus (Kützing) Kulikovskiy*	376.28	8.52	30.34	24.91	121.98
*Diadesmis confervacea* Kützing	277.47	8.17	29.57	20.44	75.08
*Encyonema minutum* (Hilse) D.G. Mann	176.05	8.23	19.02	8.58	52.04
*Encyonopsis microcephala* (Grunow) Krammer	1361.12	8.31	789.86	22.24	49.12
*Encyonopsis minuta* Krammer & E. Reichardt	630.98	8.6	262.35	12.89	73.3
*Encyonopsis subminuta* Krammer & E. Reichardt	219.96	8.52	33.45	9.92	61.23
*Craticula subminuscula* (Manguin) C.E.Wetzel & Ector	219.96	8.52	33.45	9.92	61.23
*Epithemia adnata* (Kützing) Brébisson	197.27	8.57	28.79	8.81	45.92
*Eunotia bilunaris* (Ehrenberg) Schaarschmidt	118.97	7.13	26.18	4.38	20.12
*Eunotia* *minor* (Kützing) Grunow	116.35	7.93	9.62	10.26	28.58
*Ulnaria* *biceps* (Kützing) Compère	373.64	8.67	61.58	23.42	97.91
*Fragilaria capucina* Desmazières	110.34	7.83	11.93	5.78	29.4
*Synedra nana* F.Meister	225.74	8.53	14.27	13.32	70.82
*Fragilaria tenera* (W. Smith) Lange-Bertalot	277.79	8.06	63.4	8.57	76.77
*Ulnaria acus* (Kützing) Aboal	263.88	8.39	27.97	16.76	78.61
*Fragilaria vaucheriae* (Kützing) J.B. Petersen	233.97	8.51	32.65	9.86	66.64
*Frustulia crassinervia* (Brébisson ex W.Smith) Lange-Bertalot & Krammer	1099.6	3.45	893.73	11.3	4.1
*Gomphonema* *gracile f. affine* Manguin	262.23	8.26	68.19	9.41	47.53
*Gomphonema* aff. *lagenula* Kützing	63.04	7.1	8.27	3.19	15.51
*Gomphonema angustatum* (Kützing) Rabenhorst	719.16	8.74	54.91	147.5	72.44
*Gomphonema exilissimum* (Grunow) Lange-Bertalot & E.Reichardt	353.28	7.57	100.45	13.07	39.76
*Gomphonema* *gracile* Ehrenberg	171.21	8.12	28.44	7.62	44.32
*Gomphonema lagenula* Kützing	170.29	7.98	16.46	9.26	52.59
*Gomphonema parvulum* (Kützing) Kützing	226.17	7.76	33.45	13.69	54.53
*Gomphonema saprophilum* (Lange-Bertalot & E.Reichardt) Abraca, R.Jahn, J.Zimmermann & Enke	529.55	8.66	17.02	104.14	81.52
*Gomphonema pseudoaugur* Lange-Bertalot	273.51	8.53	20	25	73.41
*Gomphonema* spp.	190.6	7.98	34.77	8.59	40.89
*Halhalamphora veneta* (Kützing) Levkov	741.37	9.42	39.73	108.43	157.87
*Mayamaea atomus* (Kützing) Lange-Bertalot	821.46	8.61	53.83	128.19	157.94
*Navicula arvensis var. dubia* Lange-Bertalot	539.45	9.45	25.57	72.12	131.35
*Navicula cryptocephala* Kützing	252.38	8.64	34.6	13.14	74.12
*Navicula radiosa* Kützing	119.67	8.2	6.59	4.41	44.76
*Navicula recens* (Lange-Bertalot) Lange-Bertalot	454.16	8.39	79.25	30.65	108.15
*Navicula rostellata* Kützing	258.42	8.43	49.08	8.98	62.01
*Navicula schroeteri* F.Meister	436.97	7.68	103.82	16.68	88.29
*Navicula* spp.	286.78	8.43	31.3	16	88.26
*Navicula trivialis* Lange-Bertalot	264.17	8.42	20.8	9.41	94.95
*Navicula veneta* Kützing	1069.75	8.5	353.02	89.54	82.81
*Navicula zanonii* Hustedt	255.84	8.61	33.59	13.02	76.15
*Nitzschia acidoclinata* Lange-Bertalot	244.25	7.69	82.73	5.9	26.4
*Nitzschia* *amphibia* Grunow	832.16	8.94	113.84	91.89	176.41
*Nitzschia archibaldii* Lange-Bertalot	250.39	8.14	43.36	11.33	63.49
*Nitzschia capitellata* Hustedt	2334.36	3.31	1438.43	12.42	2.81
*Nitzschia clausii* Hantzsch	403.33	7.18	142.48	6.29	45.26
*Nitzschia draveillensis* Coste & Ricard	326.1	7.96	31.73	24.92	94.27
*Nitzschia filiformis* (W. Smith) Van Heurck	278.6	8.69	29.81	12.12	90.59
*Nitzschia fonticola* (Grunow) Grunow	437.48	7.97	106.01	16.4	83.29
*Nitzschia frustulum* (Kützing) Grunow	183.61	8.2	19.75	6.25	61.82
*Nitzschia liebethruthii* Rabenhorst	283.4	8.14	44.09	14.15	77.96
*Nitzschia* *linearis* var. *subtilis* Hustedt	222.31	8.35	30.14	10.82	60.89
*Nitzschia* *linearis* W. Smith	354.71	8.66	50.38	8.8	104.54
*Nitzschia microcephala* Grunow	670.34	8.63	80.44	69.4	168.6
*Nitzschia* *nana* Grunow	274.53	8.43	50.28	15.33	63.54
*Nitzschia* *palea* (Kützing) W. Smith	483.7	8.47	72.12	40.29	101.6
*Nitzschia pura* Hustedt	1749.83	8.1	787.4	45.69	66.61
*Nitzschia radicula* Hustedt	165.87	8.46	17.21	7.59	51.99
*Nitzschia* *recta* Hantzsch	86.23	7.61	10.9	2.75	25.94
*Nitzschia reversa* W. Smith	265.55	8.26	48.84	10.9	50.76
*Nitzschia* spp.	330.79	8.4	86.33	10.68	62.99
*Pinnularia acrosphaeria* W. Smith	175.55	8.09	18.64	10.36	48.62
*Pinnularia divergens* W. Smith	258.5	7.94	48.56	6.04	79.6
*Pinnularia subbrevistriata* Krammer	399.76	8.81	27.43	46.65	97.82
*Pinnularia subcapitata* W. Gregory	72.78	6.99	10.03	4.04	15.94
*Pinnularia viridiformis* Krammer	228.01	7.44	44.74	9.57	65.18
*Paraplanothidium rostratum* (Østrup) Tseplik, Glushchenko, Maltsev & Kulikovskiy	261.43	8.2	27.44	14.79	77.86
*Rhopalodia gibba* (Ehrenberg) O.Müller	341.48	8.61	72.57	10.79	83.97
*Sellaphora pupula* (Kützing) Mereschkovsky	306.67	8.46	28.51	24.6	81.76
*Sellaphora seminulum* (Grunow) D.G. Mann	234.33	7.45	50.22	8.94	46.93

**Table 2:** Calculated index score per site seasons. MMI 1 was computed using equal weights for all metrics, whereas MMI 2 applied greater weights to sulphate, pH, and EC than to alkalinity or chloride

Sample ID	Site	Disturbance level observed	MMI 1	MMI 2
Mp4	1	2	25.53	20.95
Mp5	2	1	24.70	19.91
Mp11.2	3	1	24.55	20.66
Mp11.4	5	1	24.78	20.00
Mp13	6	1	24.70	20.02
Mp15	7	1	23.86	20.22
Mp16	8	2	28.16	18.29
MPW2	9	1	24.18	19.75
MPW3	10	1	25.93	20.79
MPW4	11	1	24.48	20.06
MPW4.2	12	1	23.37	18.99
MPW5	13	1	21.02	17.32
Vosstoffel	14	2	24.84	20.08
MP Ivan	15	1	23.99	19.72
Goedgevonden2/R	16	3	75.49	83.76
Goedgevonden1/L	17	3	74.63	83.38
Kendal PS	18	1	25.42	21.99
Bezhoek1	19	1	16.94	15.68
Bezhoek2	20	1	24.70	21.74
Bezhoek3	21	1	23.01	19.14
Jansen Wetland	22	1	24.21	19.63
KZN2	23	1	29.15	19.78
KZN3	24	3	29.51	21.25
KZN5	25	3	30.73	26.83
KZN6	26	1	24.21	19.78
KZN8	27	1	24.19	20.45
KZN9	28	1	21.06	14.92
KZN12	29	1	22.58	19.05
KZN17	30	3	24.61	19.88
KZN18	31	1	21.87	18.75
KZN19	32	1	23.83	20.46
KZN21	33	1	23.98	19.35
KZN36	34	3	80.33	90.04
KZN37	35	1	22.92	19.09
KZN40	36	1	21.84	17.98
KZN42	37	2	23.99	19.57
KZN47	38	1	24.23	19.70
KZN48	39	3	80.33	90.04
KZN51	40	3	27.38	21.32
KZN54	41	1	24.05	19.55
KZN56	42	1	24.71	20.31
KZN57	43	1	22.02	18.17
KZN58	44	1	23.98	20.20
WS1	45	2	24.49	21.40
WS2	46	1	20.95	17.67
Mp4	47	3	25.44	21.07
Mp5	48	1	25.98	21.60
Mp11.2	49	1	19.66	16.31
Mp11.3	50	1	20.77	17.05
Mp11.4	51	1	24.00	19.71
Mp13	52	1	23.78	19.29
Mp15	53	1	24.19	19.75
Mp16	54	1	28.64	18.93
MPW2	55	1	26.61	21.46
MPW3	56	1	26.70	21.50
MPW4	57	1	23.60	19.69
MPW4.2	58	1	23.43	19.39
MPW5	59	1	21.35	17.65
Vosstoffel	60	1	30.25	21.57
MP Ivan	61	1	23.82	19.29
Goedgevonden2/R	62	3	76.61	85.21
Goedgevonden1/L	63	3	69.53	77.40
Kendal PS	64	1	24.99	21.76
Jansen Wetland	65	1	23.45	19.15
KZN2	66	1	31.30	22.02
KZN3	67	1	33.34	22.94
KZN5	68	3	39.48	35.85
KZN6	69	1	22.99	19.77
KZN8	70	1	22.64	18.95
KZN9	71	1	23.87	19.88
KZN12	72	1	25.00	21.12
KZN17	73	3	24.88	20.18
KZN18	74	1	21.14	17.50
KZN19	75	1	22.85	18.79
KZN21	76	1	24.03	19.82
KZN36	77	4	80.33	90.04
KZN37	78	1	21.86	17.99
KZN40	79	1	23.91	19.42
KZN42	80	2	29.04	24.16
KZN47	81	1	24.34	19.77
KZN48	82	3	68.95	76.32
KZN54	83	1	23.73	19.27
KZN56	84	2	22.82	19.01
KZN57	85	1	24.17	20.22
KZN58	86	1	21.02	17.38
WS1	87	1	25.63	21.94
WS2	88	1	21.72	18.15

**Table 3:** Water quality measurements for the five environmental variables used to create the index scores, with observed disturbance levels

Sample ID	Site nr	Sulphate (mg/L)	Chloride (mg/L)	Alkalinity (mg/L)	Electrical conductivity (EC) (µS/cm)	pH	Observed disturbance level
Mp4	1	203	12	8.4	540	6.8	2
Mp5	2	30	19	43	230	9.6	1
Mp11.2	3	6.6	13	39	140	9.4	1
Mp11.4	5	8.1	24	52	210	9.6	1
Mp13	6	30	7	35	170	7.5	1
Mp15	7	12	3	7.9	60	6.7	1
Mp16	8	81	30	167	660	10	2
MPW2	9	17	10	31	150	7.9	1
MPW3	10	28	38	138	430	8.1	1
MPW4	11	38	5.6	34	190	8	1
MPW4.2	12	45	8.1	69	270	9	1
MPW5	13	29	9.4	80	230	8.9	1
Vosstoffel	14	29	88	143	620	9.7	2
MP Ivan	15	22	3.3	29	120	8.8	1
Goedgevonden2/R	16	497	2.3	1	1080	3	3
Goedgevonden1/L	17	860	2	1	1650	3.4	3
Kendal PS	18	3.8	2	48	100	7.9	1
Bezhoek1	19	2.3	2	18	50	7.4	1
Bezhoek2	20	10	2.6	10	60	6.9	1
Bezhoek3	21	3.1	3.3	6.6	30	6.8	1
Jansen Wetland	22	6.2	16	29	140	8.6	1
KZN2	23	5.3	245	70	920	9.4	1
KZN3	24	113	215	223	1300	8.5	3
KZN5	25	1468	35	35	2650	8.6	3
KZN6	26	6	2	62	140	9.6	1
KZN8	27	21	2.5	55	170	9.4	1
KZN9	28	2.5	4	107	230	9.5	1
KZN12	29	0.6	2	8.2	30	6.9	1
KZN17	30	309	8.1	69	740	9.3	3
KZN18	31	8.1	2	39	100	8.4	1
KZN19	32	10	2	4	40	6.3	1
KZN21	33	28	12	96	290	9.8	1
KZN36	34	3474	4.9	1	4750	2.8	3
KZN37	35	10	5.9	46	140	7.8	1
KZN40	36	8.2	6.4	34	120	8.7	1
KZN42	37	315	12	27	720	8.7	2
KZN47	38	16	14	135	330	8.8	1
KZN48	39	1159	33	1	1950	4.2	3
KZN51	40	1374	27	76	2650	8.2	3
KZN54	41	16	7.3	88	220	9.2	1
KZN56	42	17	4.5	45	150	8.9	1
KZN57	43	19	2.7	32	120	9	1
KZN58	44	9.6	3.2	20	80	7.1	1
WS1	45	141	9.9	1.4	370	4.7	2
WS2	46	8	4.9	34	110	8.5	1
Mp4	47	245	15	256	860	8.2	3
Mp5	48	37	26	91	350	9.1	1
Mp11.2	49	7.1	24	85	270	8.7	1
Mp11.3	50	8.2	3	35	100	8.1	1
Mp11.4	51	6.7	18	94	250	8.7	1
Mp13	52	18	13	66	220	8.7	1
Mp15	53	43	6.6	17	170	7.8	1
Mp16	54	58	77	325	980	9.5	1
MPW2	55	17	34	143	410	8.4	1
MPW3	56	9.1	25	200	450	8.7	1
MPW4	57	51	9.1	52	250	7.9	1
MPW4.2	58	38	8	84	270	8.8	1
MPW5	59	29	16	98	310	8.6	1
Vosstoffel	60	17	138	236	900	9.3	1
MP Ivan	61	39	6.7	66	250	9.6	1
Goedgevonden2/R	62	466	6.3	1	900	3	3
Goedgevonden1/L	63	650	3.1	1	1420	3.1	3
Kendal PS	64	3.3	2	155	310	7.7	1
Jansen Wetland	65	5.8	18	27	140	8.7	1
KZN2	66	7.4	325	97	1240	9	1
KZN3	67	45	111	186	820	8.7	1
KZN5	68	1160	62	52	2450	7.9	3
KZN6	69	5.2	2	102	210	9.1	1
KZN8	70	16	3.3	100	240	8	1
KZN9	71	8.9	12	82	220	8.8	1
KZN12	72	3.9	3.2	15	60	7	1
KZN17	73	575	13	55	2100	8	3
KZN18	74	7.6	2	49	120	7.7	1
KZN19	75	23	6.3	45	150	8.6	1
KZN21	76	14	6.3	87	200	7.6	1
KZN36	77	3534	8.7	1	7400	2.5	4
KZN37	78	6.5	3.4	50	120	8.8	1
KZN40	79	4.2	8.5	46	130	9.1	1
KZN42	80	141	13	67	580	8.7	2
KZN47	81	2.4	21	86	300	9.2	1
KZN48	82	1406	33	1	210	3.6	3
KZN54	83	12	11	84	210	9.3	1
KZN56	84	97	8	28	300	7.2	2
KZN57	85	23	4.7	38	140	9.4	1
KZN58	86	11	6.8	28	110	8.9	1
WS1	87	8.7	7.6	40	130	8.5	1
WS2	88	3.6	2.7	37	90	8	1

**Table 4:** Wetland types for sites sampled with corresponding disturbance levels and GPS coordinates

Site nr	Sample ID	Season	Colour code	Type	Abbreviation	Obesrved disturbance level	Ref/Dist	Location
1	Mp4	Dry	Purple	Seep	S	2	D	−25.9512356424, 29.156113379
2	Mp5	Dry	Purple	Seep	S	1	R	−25.9750832714, 29.150626362
3	Mp11.2	Dry	Orange	Channelled valley-bottom wetland	CVBW	1	R	−25.8151519447, 29.7950744621
5	Mp11.4	Dry	Purple	Seep	S	1	R	−25.8596233396, 29.810679921
6	Mp13	Dry	Purple	Seep	S	1	R	−26.1177090148, 29.0118737622
7	Mp15	Dry	Turquoise	Wetland flat	WF	1	R	−26.0785114235, 29.0206846542
8	Mp16	Dry	Turquoise	Wetland flat	WF	2	D	−26.1021753466, 28.9964742911
9	MPW2	Dry	Orange	Channelled valley-bottom wetland	CVBW	1	R	−26.291683, 29.084064
10	MPW3	Dry	Orange	Channelled valley-bottom wetland	CVBW	1	R	−26.268247, 29.00955
11	MPW4	Dry	Purple	Seep	S	1	R	−26.206667, 29.012133
12	MPW4.2	Dry	Purple	Seep	S	1	R	−26.206667, 29.012133
13	MPW5	Dry	Orange	Channelled valley-bottom wetland	CVBW	1	R	−26.157328, 28.94608
14	Vosstoffel	Dry	Turquoise	Wetland flat	WF	2	D	−26.41675, 29.13209
15	MP Ivan	Dry	Orange	Channelled valley-bottom wetland	CVBW	1	R	−26.0958556, 29.0088861
16	Goedgevonden2/R	Dry	Orange	Channelled valley-bottom wetland	CVBW	3	D	−26.060364, 29.0710583
17	Goedgevonden1/L	Dry	Orange	Channelled valley-bottom wetland	CVBW	3	D	−26.0590028, 29.0662611
18	Kendal PS	Dry	Blue	Depression	D	1	R	−26.0622196501, 28.9540035389
19	Bezhoek1	Dry	Orange	Channelled valley-bottom wetland	CVBW	1	R	−25.7425111, 29.3310778
20	Bezhoek2	Dry	Orange	Channelled valley-bottom wetland	CVBW	1	R	−25.7461611, 29.3300417
21	Bezhoek3	Dry	Yellow	Unchanneled valley-bottom wetland	UVBW	1	R	−25.7446056, 29.324675
22	Jansen Wetland	Dry	Yellow	Unchanneled valley-bottom wetland	UVBW	1	R	−25.6572333, 29.5670361
23	KZN2	Dry	Purple	Seep	S	1	R	−27.8542994401, 30.1679377028
24	KZN3	Dry	Blue	Depression	D	3	D	−27.639516997, 30.1268284946
25	KZN5	Dry	Pink	Floodplain wetland	FPW	3	D	−27.6098621623, 30.1494935529
26	KZN6	Dry	Orange	Channelled valley-bottom wetland	CVBW	1	R	−27.6142183052, 30.1140342803
27	KZN8	Dry	Orange	Channelled valley-bottom wetland	CVBW	1	R	−27.6117969292, 30.1331093533
28	KZN9	Dry	Green	Valley head seep	VHS	1	R	−27.6294036438, 30.1350810576
29	KZN12	Dry	Turquoise	Wetland flat	WF	1	R	−27.8819842443, 30.1553308577
30	KZN17	Dry	Turquoise	Wetland flat	WF	3	D	−27.8515660356, 30.1091353401
31	KZN18	Dry	Purple	Seep	S	1	R	−27.8523252468, 30.0987325487
32	KZN19	Dry	Purple	Seep	S	1	R	−27.8529105653, 30.1002134047
33	KZN21	Dry	Purple	Seep	S	1	R	−27.8563880915, 30.0977134068
34	KZN36	Dry	Green	Valley head seep	VHS	3	D	−28.0564937359, 30.1319930023
35	KZN37	Dry	Turquoise	Wetland flat	WF	1	R	−28.0660219083, 30.1261468652
36	KZN40	Dry	Orange	Channelled valley-bottom wetland	CVBW	1	R	−28.0838647893, 30.1494043745
37	KZN42	Dry	Orange	Channelled valley-bottom wetland	CVBW	2	D	−28.0475199785, 30.1680241634
38	KZN47	Dry	Purple	Seep	S	1	R	−28.0927716877, 30.1869078163
39	KZN48	Dry	Yellow	Unchanneled valley-bottom wetland	UVBW	3	D	−28.0608365398, 30.0144388433
40	KZN51	Dry	Orange	Channelled valley-bottom wetland	CVBW	3	D	−28.0654720064, 29.9698637072
41	KZN54	Dry	Orange	Channelled valley-bottom wetland	CVBW	1	R	−28.0821613332, 30.013674451
42	KZN56	Dry	Purple	Seep	S	1	R	−28.0430131659, 30.0190240659
43	KZN57	Dry	Purple	Seep	S	1	R	−28.0390809322, 30.017521668
44	KZN58	Dry	Purple	Seep	S	1	R	−28.0423214149, 30.0005792695
45	WS1	Dry	Pink	Floodplain wetland	FPW	2	D	−27.3643316027, 30.1275673504
46	WS2	Dry	Orange	Channelled valley-bottom wetland	CVBW	1	R	−27.3318655572, 30.1586151225
47	Mp4	Wet	Purple	Seep	S	3	D	−25.9512356424, 29.156113379
48	Mp5	Wet	Purple	Seep	S	1	R	−25.9750832714, 29.150626362
49	Mp11.2	Wet	Orange	Channelled valley-bottom wetland	CVBW	1	R	−25.8151519447, 29.7950744621
50	Mp11.3	Wet	Orange	Channelled valley-bottom wetland	CVBW	1	R	−25.8463981331, 29.801032113
51	Mp11.4	Wet	Purple	Seep	S	1	R	−25.8596233396, 29.810679921
52	Mp13	Wet	Purple	Seep	S	1	R	−26.1177090148, 29.0118737622
53	Mp15	Wet	Turquoise	Wetland flat	WF	1	R	−26.0785114235, 29.0206846542
54	Mp16	Wet	Turquoise	Wetland flat	WF	1	R	−26.1021753466, 28.9964742911
55	MPW2	Wet	Orange	Channelled valley-bottom wetland	CVBW	1	R	−26.291683, 29.084064
56	MPW3	Wet	Orange	Channelled valley-bottom wetland	CVBW	1	R	−26.268247, 29.00955
57	MPW4	Wet	Purple	Seep	S	1	R	−26.206667, 29.012133
58	MPW4.2	Wet	Purple	Seep	S	1	R	−26.206667, 29.012133
59	MPW5	Wet	Orange	Channelled valley-bottom wetland	CVBW	1	R	−26.157328, 28.94608
60	Vosstoffel	Wet	Turquoise	Wetland flat	WF	1	R	−26.41675, 29.13209
61	MP Ivan	Wet	Orange	Channelled valley-bottom wetland	CVBW	1	R	−26.0958556, 29.0088861
62	Goedgevonden2/R	Wet	Orange	Channelled valley-bottom wetland	CVBW	3	D	−26.060364, 29.0710583
63	Goedgevonden1/L	Wet	Orange	Channelled valley-bottom wetland	CVBW	3	D	−26.0590028, 29.0662611
64	Kendal PS	Wet	Blue	Depression	D	1	R	−26.09913, 28.96575
65	Jansen Wetland	Wet	Yellow	Unchanneled valley-bottom wetland	UVBW	1	R	−25.6572333, 29.5670361
66	KZN2	Wet	Purple	Seep	S	1	R	−27.8542994401, 30.1679377028
67	KZN3	Wet	Blue	Depression	D	1	R	−27.639516997, 30.1268284946
68	KZN5	Wet	Pink	Floodplain wetland	FPW	3	D	−27.6098621623, 30.1494935529
69	KZN6	Wet	Orange	Channelled valley-bottom wetland	CVBW	1	R	−27.6142183052, 30.1140342803
70	KZN8	Wet	Orange	Channelled valley-bottom wetland	CVBW	1	R	−27.6117969292, 30.1331093533
71	KZN9	Wet	Green	Valley head seep	VHS	1	R	−27.6294036438, 30.1350810576
72	KZN12	Wet	Turquoise	Wetland flat	WF	1	R	−27.8819842443, 30.1553308577
73	KZN17	Wet	Turquoise	Wetland flat	WF	3	D	−27.8515660356, 30.1091353401
74	KZN18	Wet	Purple	Seep	S	1	R	−27.8523252468, 30.0987325487
75	KZN19	Wet	Purple	Seep	S	1	R	−27.8529105653, 30.1002134047
76	KZN21	Wet	Purple	Seep	S	1	R	−27.8563880915, 30.0977134068
77	KZN36	Wet	Green	Valley head seep	VHS	4	D	−28.0564937359, 30.1319930023
78	KZN37	Wet	Turquoise	Wetland flat	WF	1	R	−28.0660219083, 30.1261468652
79	KZN40	Wet	Orange	Channelled valley-bottom wetland	CVBW	1	R	−28.0838647893, 30.1494043745
80	KZN42	Wet	Orange	Channelled valley-bottom wetland	CVBW	2	D	−28.0475199785, 30.1680241634
81	KZN47	Wet	Purple	Seep	S	1	R	−28.0927716877, 30.1869078163
82	KZN48	Wet	Yellow	Unchanneled valley-bottom wetland	UVBW	3	D	−28.0608365398, 30.0144388433
83	KZN54	Wet	Orange	Channelled valley-bottom wetland	CVBW	1	R	−28.0821613332, 30.013674451
84	KZN56	Wet	Purple	Seep	S	2	D	−28.0430131659, 30.0190240659
85	KZN57	Wet	Purple	Seep	S	1	R	−28.0390809322, 30.017521668
86	KZN58	Wet	Purple	Seep	S	1	R	−28.0423214149, 30.0005792695
87	WS1	Wet	Pink	Floodplain wetland	FPW	1	R	−27.3643316027, 30.1275673504
88	WS2	Wet	Orange	Channelled valley-bottom wetland	CVBW	1	R	−27.3318655572, 30.1586151225
